# The Chinese Version of the DigiHealthCom (Digital Health Competence) Instrument for Assessing Digital Health Competence of Health Care Professionals: Translation, Adaptation, and Validation Study

**DOI:** 10.2196/65373

**Published:** 2025-03-21

**Authors:** Lu Gao, Meilian Chen, Jingxin Wei, Jinni Wang, Xiaoyan Liao

**Affiliations:** 1 School of Nursing, Southern Medical University, Guangzhou, China; 2 Nanfang Hospital, Southern Medical University, Guangzhou, China; 3 School of Nursing, Guangzhou Medical University, Panyu Campus, No. 1 Xinzao Road, Panyu District, Guangzhou, 511436, China, 86 18665039967, 86 2061641917

**Keywords:** competence, digital health, health care professionals, instrument, reliability, validity

## Abstract

**Background:**

Digital health competence is increasingly recognized as a core competence for health care professionals. A comprehensive evaluation of knowledge, skills, performance, values, and attitudes necessary to adapt to evolving digital health technologies is essential. DigiHealthCom (Digital Health Competence) is a well-established instrument designed to assess digital health competence across diverse health care professionals.

**Objective:**

This study aimed to translate and culturally adapt DigiHealthCom into simplified Chinese (Mandarin) and verify its reliability and validity in assessing digital health competence of Chinese health care professionals.

**Methods:**

DigiHealthCom was translated into Chinese following the guideline proposed by its original developers. The cultural adaptation involved expert review and cognitive interviewing. Internal consistency, test-retest reliability, content validity, convergent validity, discriminant validity, and factor structure were examined. Item analysis tested item discrimination, item correlation, and item homogeneity. Internal consistency was assessed using Cronbach α, and test-retest reliability was measured using the intraclass correlation coefficient. Content validity was assessed through both item and scale content validity indices. Convergent validity was measured by the Average Variance Extracted and Composite Reliability, while discriminant validity was measured by the heterotrait-monotrait ratio. A five-dimension model of DigiHealthCom was confirmed using confirmatory factor analysis.

**Results:**

The finalized Chinese version of the DigiHealthCom was completed after addressing differences between the back-translations and the original version. No discrepancies affecting item clarity were reported during cognitive interviewing. The validation process involved 398 eligible health care professionals from 36 cities across 15 provinces in China, with 43 participants undergoing a retest after a 2-week interval. Critical ratio values (range 16.05‐23.77, *P*<.001), item-total correlation coefficients (range 0.69‐0.89), and Cronbach α if the item deleted (range 0.91‐0.96) indicated satisfactory item discrimination, item correlation, and item homogeneity. Cronbach α for dimensions and the scale ranged from 0.94 to 0.98, indicating good internal consistency. The intraclass correlation coefficient was 0.90 (95% CI 0.81‐0.95), indicating good test-retest reliability. Item content validity index ranged from 0.82 to 1.00, and the scale content validity index was 0.97, indicating satisfactory content validity. Convergent validity (average variance extracted: 0.60‐0.79; composite reliability: 0.94‐0.95) and divergent validity (heterotrait-monotrait ratio: 0.72‐0.89) were satisfactory. Confirmatory factor analysis confirmed a well-fit five-dimension model (robust chi-square to df ratio=3.10, comparative fit index=0.91, Tucker-Lewis index=0.90, incremental fit index=0.91, root-mean-square error of approximation=0.07, standardized root-mean-square residual=0.05), with each item having a factor loading exceeding 0.40.

**Conclusions:**

The Chinese version of DigiHealthCom has been proved to be reliable and valid. It is now available for assessing digital health competence among Chinese health care professionals. This assessment can be used to guide health care policy makers and educators in designing comprehensive and implementable educational programs and interventions.

## Introduction

The World Health Organization (WHO) defines digital health as the field that involves the development and use of digital technologies to improve health outcomes [1]. In 2019, the WHO released the world’s first guidelines for digital health interventions, outlining 10 ways to use digital health technologies to enhance health and primary services [2]. This concept extends beyond eHealth to include a wide array of smart devices and connected equipment, encompassing digital technologies such as the Internet of Things, big data, artificial intelligence, and robotics [2]. The release of these guidelines was followed by the Global Strategy for Digital Health (2020‐2025), which emphasizes prioritized digital health strategies for global health care development [1]. China has incorporated measures for the development of a digitally healthy nation in the 14th Five-Year Plan [3]. With the global market projected to grow from US $211 billion in 2022 to US $809.2 billion by 2030 [4], digital health care is recognized as a rapidly expanding sector. Digital health has emerged as a significant trend in the evolution of global health care services. The digital transformation that the health care sector is currently undergoing is redefining the roles and responsibilities of health care professionals [5,6], creating an urgent need for digital health competence among them.

Given increasing prominence of digital health in global health care landscape, it is critical for health care professionals to possess sufficient digital health competence. Digital health competence is increasingly recognized as one of the core competencies for health care professionals, which would enable them to design and evaluate the impact of digital solutions on patient care and determine the best way to implement digital solutions in their work [7,8]. Although patients are becoming more accepting of and motivated to use digital health care services and tools, health care professionals face a digital skills shortage that impedes the adoption of digital solutions [9-11]. Inadequate digital health competence may lead to negative experiences and frustration with technology adoption among these professionals [12]. There is a strong association between health care professionals’ digital health competence and their willingness to use such tools [13,14]. Studies have highlighted that the acceptance of digital health technologies by health care professionals significantly influences the adoption of digital solutions and emphasizes the critical role of digital health competence in ensuring patient safety [12,14]. Therefore, assessing digital health competence is essential to effectively providing digital health care solutions to the public.

Previous studies have attempted to explore digital health competence among health care professionals but have struggled to define it comprehensively due to the evolving nature of digital technologies [15]. Existing assessment tools primarily focus on informatics competence, digital health literacy, or skills related to the application of digital technologies. Examples include the Digital Health Literacy Instrument (DHLI, 2017) [16], the eHealth literacy questionnaire (eHLQ, 2018) [17], and the Nursing Digital Application Skill Scale (NDASS, 2024) [3]. The eHLQ is designed for eHealth user, especially individuals with low digital health literacy and those with chronic conditions [17]. The NDASS targets nurses’ digital application skills in clinical settings [3]. Digital health literacy reflects users’ knowledge and skills within their cultural, social, and institutional context [17]. Competence entails an integrative understanding of the knowledge, skills, performance, values, and attitudes essential for the effective execution of a given task [18]. Therefore, it is crucial to comprehensively evaluate the knowledge, skills, performance, and attitudes required for various health care professionals to adapt to the evolving digital health technologies.

Developed and validated among Finnish health care professionals in 2022, the DigiHealthCom (Digital Health Competence) instrument offers a more comprehensive scope than existing tools and is applicable to a wide range of health care professionals. In addition to assessing competence in using digital solutions and information and communication technology (ICT), it also explores previously unaddressed domains that reflect future requirements, such as acceptance of digital solutions, human-centered remote counseling, and ethical competence concerning digital solutions [19]. Furthermore, the instrument has been used to explore digital health competence profiles and associated factors in 817 health care professionals from 9 organizations in Finland [20]. It has been translated into 15 languages, and a large-scale international cross-sectional study on the digital health competence of health care professionals is currently in progress. Our team is part of this collaborative research effort. This study aimed to culturally adapt and validate the Chinese version of DigiHealthCom for Chinese health care professionals.

## Methods

### Study Design

A cross-sectional study was conducted.

### Participants

Participants were recruited between May 2023 and April 2024 via convenience sampling. Recruitment posters with QR codes were disseminated on social networks specific to health care professionals. In addition, health care professionals attending local academic conferences were invited to participate. The inclusion criteria for participants were (1) employment within a health care organization and (2) consent to participate. The exclusion criteria were (1) individuals who were retired or had less than 1 year of work experience; (2) health care students; (3) individuals who completed the questionnaire in less than 150 seconds, as this indicated random clicking; or (4) individuals who displayed erratic response patterns.

According to the thumb rule, the sample size should be 5 to 10 times the number of items. With an anticipated 10% rate of invalid responses, a minimum of 231 samples was necessary. Finally, a total of 398 cases were included in the study. To assess the test-retest reliability of the instrument, 43 participants who provided contact information completed the questionnaire again after a 2-week interval.

### Instrument

The DigiHealthCom instrument comprises 5 domains, comprising a total of 42 items—competence in human-centered remote counseling (16 items), digital solutions as part of work (9 items), competence in ICT (5 items), competence in using and evaluating digital solutions (8 items), and ethical competence related to digital solutions (4 items). Each item was rated on a 4-point Likert scale (1=completely disagree, 2=partially disagree, 3=partially agree, and 4=completely agree). For each domain, a mean value of ≤2.49 indicated low competence, 2.50‐3.49 indicated intermediate competence, and ≥3.50 indicated high competence [20]. DigiHealthCom has been validated among health care professionals across tertiary, primary, and private health care settings (n=817), demonstrating satisfactory internal consistency (Cronbach α=0.80‐0.97) and content validity (item content validity index [I-CVI]: 0.77‐1.00; S-CVI/Ave: 0.94) [19].

#### Translation and Cross-Cultural Adaptation

**Figure 1.**
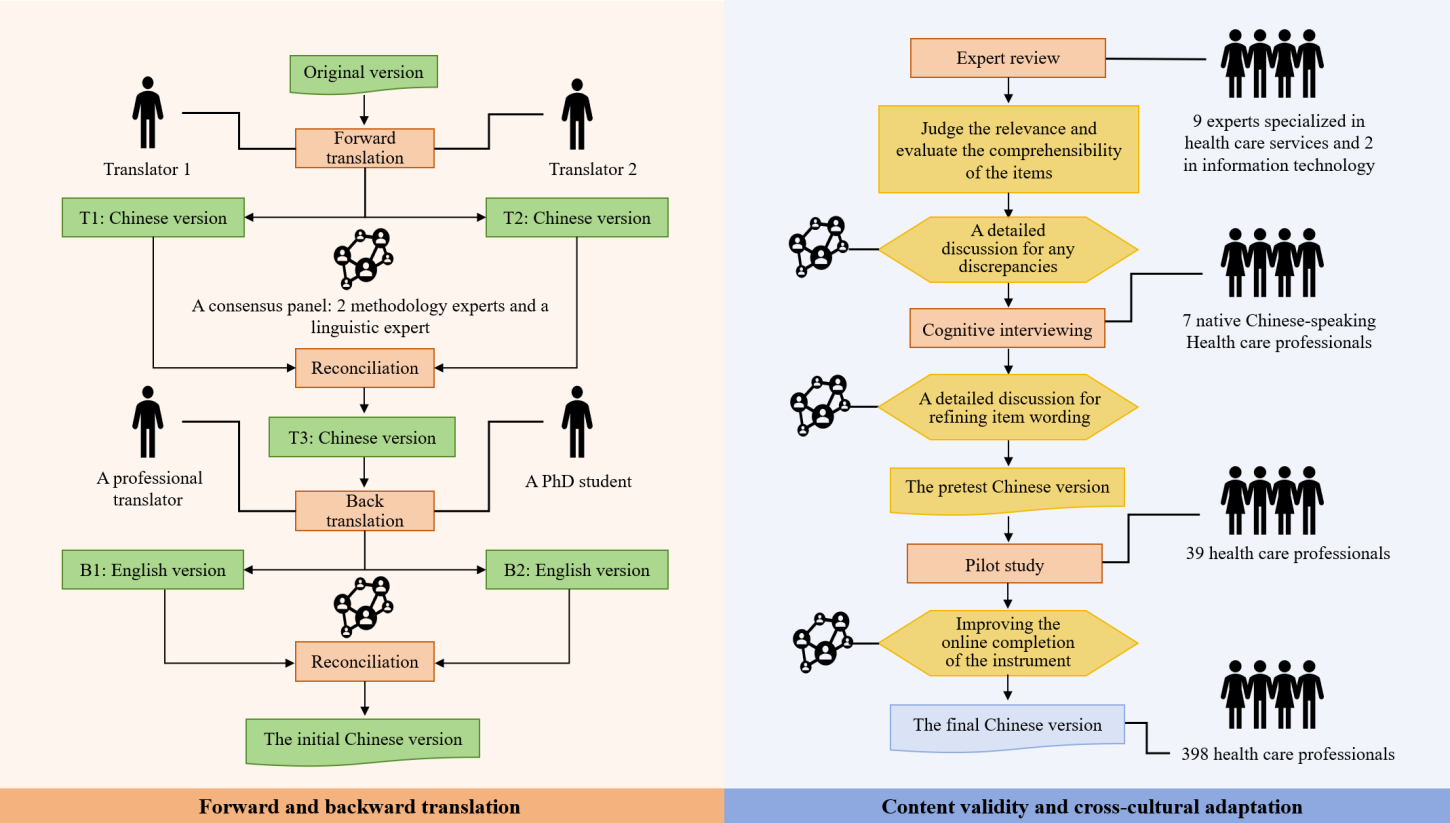
The translation and cross-cultural adaptation process of developing the Chinese version of the DigiHealthCom (Digital Health Competence) instrument.

To ensure a high-quality Chinese translation of DigiHealthCom, a rigorous translation and cross-cultural adaptation process was followed [21]. Figure 1 illustrates the translation and cross-cultural adaptation process.

#### Forward and Backward Translation

In total, 2 bilingual nursing professionals, who are native Chinese speakers (a nursing expert and a nursing graduate student), independently translated the English version of DigiHealthCom into Chinese, resulting in 2 forward translations (T1 and T2). A consensus panel reviewed these translations for conceptual equivalence, clarity, and comprehensibility, producing a reconciled version (T3). Subsequently, the T3 version was back-translated into English by a professional translator and a PhD student unfamiliar with the original version, both knowledgeable about Chinese and English-speaking cultures. The consensus panel reviewed and resolved discrepancies between the back-translations and the original version, finalizing the initial Chinese version. The consensus panel comprised 2 nursing researchers experienced in scale development and a linguistic expert.

#### Expert Review

To assess the content validity of the Chinese version of DigiHealthCom, 11 experts were invited to evaluate the relevance of its dimensions and items using a 4-point ordinal scale (1 [not relevant], 2 [weakly relevant], 3 [strongly relevant], and 4 [very relevant]). The expert panel consisted of 9 health care specialists and 2 IT experts. In addition, expert evaluated the comprehensibility of the items. The consensus panel, initially involved in the translation process, conducted a detailed discussion to resolve any potential discrepancies.

#### Cognitive Interviewing

Cognitive interviewing was used to evaluate the clarity and cultural suitability of the initial Chinese version. A total of 7 native Chinese speakers, including 2 doctors, 4 nurses, and 1 IT technician, were recruited. Participants were briefed on the study’s objectives and methods before interviews, and their consent was obtained.

The first author, trained in qualitative research methods, conducted the interviews in a meeting room. Participants completed the Chinese version of DigiHealthCom independently and engaged in cognitive interviews. Interviewers evaluated whether participants found the items relevant to their condition and if they encountered any understanding difficulties. Field notes were taken. Modifications were deemed necessary if at least 1 participant (1) found an item difficult to understand, (2) demonstrated mostly or completely inaccurate comprehension of an item, or (3) provided feedback indicating the need for improvements, especially regarding cultural relevance. The consensus panel determined whether to retain or alter item wording following expert review and cognitive interviewing. Discrepancies affecting item clarity were resolved to create the pretest version of the Chinese DigiHealthCom version. Specific item modifications were detailed in Table S1 in Multimedia Appendix 1.

#### Pilot Study

A pilot study was conducted with 39 health care professionals from a general hospital in Guangzhou, China. The pilot study confirmed that no further modifications were required. The final Chinese version of DigiHealthCom consists of 5 dimensions and 42 items.

### Data Collection

Data were collected using the Wen Juan Xing (a Chinese web questionnaire platform) [22]. Respondents accessed the questionnaire by scanning a QR code or clicking a link, with 1 response allowed per IP address to prevent duplicates. The questionnaire platform performed completeness checks before submission. The Chinese version of DigiHealthCom was presented alongside a sociodemographic questionnaire. The questionnaire consisted of 2 pages and 73 items. Sex, age, education, service area, professional license, work experience, type of organization, and frequency of patient work were collected. Participants who voluntarily chose to provide contact information participated in a retest after a 2-week interval. An introduction outlined the study’s purpose and provided questionnaire instructions.

### Statistical Analysis

Item analysis was used to evaluate item discrimination, item correlation, and item homogeneity. For item discrimination, respondents were classified into high-score (top 27%) and low-score (bottom 27%) groups based on their total scores. An independent *t* test determined whether each item could significantly distinguish between these groups. Items with a critical ratio (|*t*|) less than 3.0 were considered for exclusion [23]. In addition, item homogeneity and item correlation were tested using Cronbach α if the item was deleted and corrected item-total correlation coefficient. Items with a corrected item-total correlation coefficient below 0.40 were considered for exclusion [23]. Cronbach α and Intraclass correlation coefficient (ICC) were used to assess the internal consistency and test-retest reliability of the instrument. A Cronbach α≥0.70 indicated good internal consistency, while ICC>0.70 indicated good time stability [23]. The I-CVI and the Scale CVI/Average (S-CVI/Ave) were used to evaluate content validity of the instrument. I-CVI≥0.78 and S-CVI/Ave≥0.90 indicate satisfactory content validity [24].

Construct validity was evaluated using confirmatory factor analysis (CFA) with a robust chi-square to df ratio (*χ*^2^*/df*) less than 3, Tucker-Lewis Index (TLI), Incremental Fit Index (IFI), and Comparative Fit Index (CFI) greater than 0.90, and root-mean-square error of approximation (RMSEA) and standardized root-mean-square residual (SRMR) less than 0.08, indicating an acceptable data-model fit [25]. The average variance extracted (AVE) and composite reliability (CR) were used to assess convergent validity, with AVE greater than 0.50 and CR greater than 0.70 indicating good convergent validity [26]. Discriminant validity was performed using the heterotrait-monotrait ratio (HTMT), with a correlation matrix value <0.90 considered good [26].

Furthermore, participants were divided into 2 groups based on geographic location for the sensitivity analysis. The DigiHealthCom scores were compared to evaluate potential selection bias. The absence of a significant difference in digital health competence between the groups indicates that selection bias is unlikely in the study sample. Statistical analysis was conducted using IBM SPSS (version 27.0), IBM AMOS (version 29.0), and Smart PLS (Version 4.1.0.0; GmbH Corp). A *P* value of less than .05 was considered statistically significant.

### Ethical Considerations

Ethical approval was obtained from the Ethical Committee at Nanfang Hospital, Southern Medical University, China (NFEC-2023‐165). This research adhered to the principals of the Declaration of Helsinki. All participants provided informed consent and voluntarily completed the web questionnaire. Participants had the option to skip questions, review, and delete their responses. Participants had the right to withdraw from the survey at any time. Those who completed it received a random monetary reward ranging from CNY 2 to 5 (US $0.28 to 0.69). Participants’ rights and researcher’s contact information were provided on the first page of the web survey. Minimal sociodemographic was collected to maintain ethical standards. Participant information was kept confidential and anonymous. The CHERRIES (Checklist for Reporting Results of Internet E-Surveys; Checklist 1) was used to enhance the transparency of the study [27].

## Results

### Characteristics of the Participants

**Figure 2.**
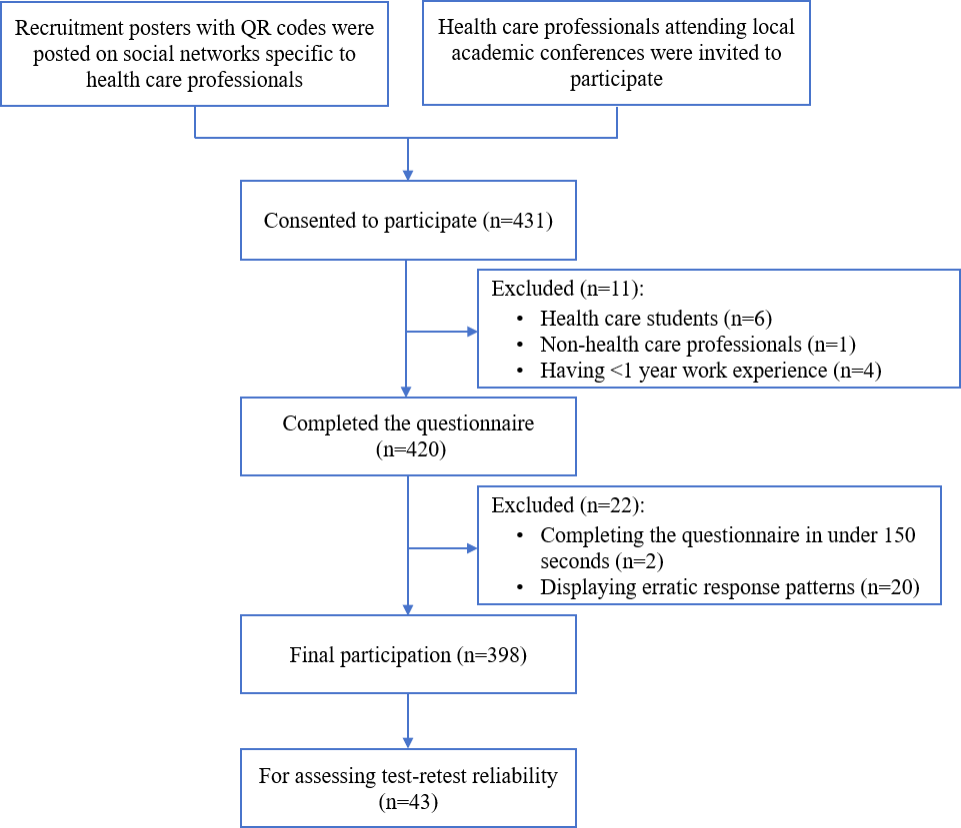
The flowchart for the enrollment process of health care professionals.

Initially, 431 participants were recruited for this study. Furthermore, 33 participants were subsequently excluded due to not meeting the inclusion criteria (n=7), having less than 1 year work experience (n=4), completing the questionnaire in under 150 seconds (n=2), and exhibiting erratic response patterns (n=20). Finally, 398 eligible health care professionals were included. Figure 2 illustrates the participant enrollment flowchart. The participants enrolled from 36 cities across 15 provinces, as identified by IP addresses. Among the participants, 249 (62.6%) were recruited from Guangzhou, a megacity in Guangdong province that contains 6125 registered medical facilities. As shown in Table 1, 364 (91.5%) participants were female, 386 (97%) worked in health care services, and 357 (89.7%) were nurses. The participants had an average of 13.7 (SD 9.4) years of work experience, with 281 (70.6%) working directly with patients for at least 5 days per week. Participant characteristics are summarized in Table 1.

**Table 1.** Demographic characteristics of the participants (N=398).

Variables		Value
**Sex, n (%)**		
	Female	364 (91.5)
	Male	34 (8.5)
Age (years), mean (SD)		36.1 (8.8)
**Education, n (%)**		
	Junior vocational qualification	58 (14.6)
	Bachelor’s degree	273 (68.6)
	Master’s degree	53 (13.3)
	Doctoral degree	14 (3.5)
**Service area, n (%)**		
	Health care service	386 (97)
	Social service	4 (1)
	Rehabilitation service	7 (1.8)
	Others	1 (0.3)
**Location, n (%)**		
	Southern China	351 (88.2)
	Northern and Western China	47 (11.8)
**Type of organization, n (%)**		
	Tertiary hospital	248 (62.3)
	Secondary hospital	36 (9)
	Community health care center	76 (19.1)
**Professional license, n (%)**		
	Nurse	357 (89.7)
	Doctor	24 (6)
	Midwife	10 (2.5)
	Others^a^	7 (1.8)
Working experience (years), mean (SD)		13.7 (9.4)
**Patient work, n (%)**		
	Daily (at least 5 days a week)	281 (70.6)
	Weekly (1-4 days per week)	62 (15.6)
	Monthly (a few times a month)	17 (4.3)
	Rarely (a few times in several months)	23 (5.8)
	I do not currently work with patient	15 (3.8)
Full-time, n (%)		398 (100)

a Including physiotherapist, paramedical technician, and pharmacist.

### Results of Item Analysis

Item analysis showed a significant difference between high-score and low-score groups. The critical ratio values for all items were above 3.0 (range 16.05‐23.77, *P*<.001; Table 2), indicating excellent item discrimination. All corrected item-total correlation coefficients exceeded 0.4 (Table 2). The Cronbach α if the item deleted (range 0.91‐0.96) were acceptable (Table 2).

**Table 2.** Item analysis and content validity of the 42 items in the DigiHealthCom (Digital Health Competence; N=398).

Item	Critical ratio	CITC^a^	CID^b^	I-CVI^c^
RC1^d^	16.05	0.69	0.96	1
RC2	17.27	0.70	0.96	1
RC3	17.29	0.71	0.96	1
RC4	19.43	0.77	0.96	1
RC5	17.10	0.77	0.96	1
RC6	20.34	0.79	0.96	1
RC7	19.90	0.80	0.96	1
RC8	22.99	0.79	0.96	0.82
RC9	19.33	0.76	0.96	1
RC10	21.91	0.81	0.96	1
RC11	19.26	0.76	0.96	1
RC12	20.84	0.80	0.96	0.91
RC13	19.89	0.80	0.96	1
RC14	20.80	0.74	0.96	1
RC15	19.02	0.73	0.96	0.91
RC16	18.71	0.75	0.96	0.91
DS1^e^	21.53	0.77	0.96	1
DS2	20.63	0.81	0.95	1
DS3	19.82	0.84	0.95	1
DS4	21.53	0.86	0.95	1
DS5	22.19	0.89	0.95	0.91
DS6	22.00	0.86	0.95	1
DS7	22.57	0.87	0.95	0.91
DS8	20.62	0.71	0.96	0.91
DS9	23.77	0.81	0.95	0.91
ICT1^f^	20.62	0.84	0.92	1
ICT2	18.64	0.88	0.91	1
ICT3	22.03	0.88	0.91	1
ICT4	18.67	0.81	0.93	1
ICT5	18.88	0.75	0.94	0.82
UE1^g^	20.48	0.82	0.95	1
UE2	21.89	0.82	0.95	1
UE3	23.04	0.84	0.95	1
UE4	22.25	0.87	0.95	0.91
UE5	21.33	0.83	0.95	1
UE6	18.19	0.78	0.95	0.91
UE7	23.55	0.87	0.95	0.91
UE8	20.52	0.82	0.95	1
EC1^h^	21.40	0.84	0.92	1
EC2	20.79	0.81	0.93	0.91
EC3	20.48	0.89	0.91	1
EC4	21.44	0.86	0.91	1

a CITC: corrected item-total correlation.

b CID: Cronbach α if item deleted.

c I-CVI: item content validity index.

d RC: human-centered remote counseling competence.

e DS: digital solutions as part of work.

f ICT: information and communication technology competence.

g UE: competence in using and evaluating digital solutions.

h EC: ethical competence related to digital solution.

### Internal Consistency and Test-Retest Reliability

As shown in Table 3, internal consistency (Cronbach α=0.94‐0.98) and test-retest reliability were good (ICC=0.90; 95% CI 0.81‐0.95).

**Table 3.** Internal consistency and test-retest reliability of the Chinese version of the DigiHealthCom (Digital Health Competence).

Dimension	Cronbach α (N=398)	ICC^a^ (N=43)	95% CI of ICC
RC^b^	0.96	0.80	0.63-0.89
DS^c^	0.96	0.90	0.81-0.95
ICT^d^	0.94	0.79	0.62-0.89
UE^e^	0.96	0.80	0.63-0.89
EC^f^	0.94	0.81	0.65-0.90
Total	0.98	0.90	0.81-0.95

a ICC: intraclass correlation coefficient.

b RC: human-centered remote counseling competence.

c DS: digital solutions as part of work.

d ICT: information and communication technology competence.

e UE: competence in using and evaluating digital solutions.

f EC: ethical competence related to digital solutions.

### Content Validity

To test content validity, 11 experts evaluated the relevance of the items. The S-CVI/Ave for the instrument was 0.97, and the I-CVI ranged from 0.82 to 1.00 (Table 2), indicating satisfactory content validity.

### Construct Validity

**Figure 3.**
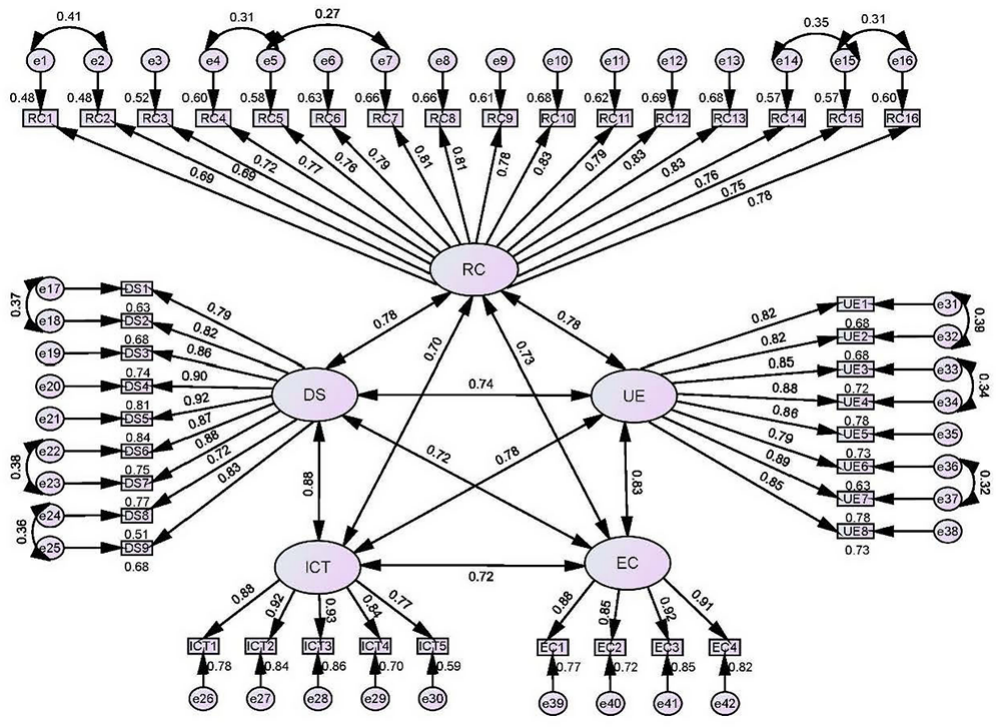
The confirmatory factor model of the Chinese version of the DigiHealthCom (Digital Health Competence; N=398). DS: digital solutions as part of work; EC: ethical competence related to digital solutions; ICT: information and communication technology competence; RC: human-centered remote counselling competence; UE: competence in using and evaluating digital solutions.

As illustrated in Figure 3, the results of CFA supported the 5-factors structure (digital solutions as part of work; ethical competence related to digital solutions; ICT competence; human-centered remote counselling competence; competence in using and evaluating digital solutions). The indices, including *χ^2^/df* (3.10), CFI (0.91), TLI (0.90), IFI (0.91), RMSEA (0.07), and SRMR (0.05), indicated an acceptable model fit. The factor loadings of items ranged from 0.69 to 0.93 (Figure 3). The AVE values for each dimension exceeded 0.50 (range: 0.60‐0.79), with the CR values >0.70 (range: 0.94‐0.95), indicating excellent convergent validity. The HTMT values within the matrix were less than 0.90 (range: 0.72‐0.89), indicating good divergent validity. These results collectively demonstrated satisfactory construct validity of the instrument.

### Sensitivity Analysis

Participants were divided into 2 groups based on geographic location: Southern China and Northern and Western China. The DigiHealthCom scores were compared to evaluate potential selection bias. As illustrated in Table S3 in Multimedia Appendix 2, no significant difference was found in DigiHealthCom scores between the 2 groups (*P*>.05), indicating that selection bias is unlikely in the study sample.

## Discussion

### Principal Findings

In this study, we translated and culturally adapted the DigiHealthCom instrument into Chinese and assessed its reliability and validity among Chinese health care professionals. The Chinese version of DigiHealthCom demonstrated satisfactory internal consistency, test-retest reliability, content validity, and construct validity. To our knowledge, this study represents the first effort to validate a comprehensive Chinese tool for measuring digital health competence among health care professionals.

In this study, each item of the Chinese version of DigiHealthCom was translated and back-translated strictly following the dual direct-to-back translation model [21] to ensure alignment in semantic, conceptual, and content with the original English version. And then, we conducted expert review involving 11 experts in nursing and IT to examine content validity of the Chinese version. High content validity indices imply that the instrument provides a broad enough range of content to allow conclusions about the targeted construct. We also conducted cognitive interviewing and a pilot study to ensure its clarity and comprehensibility [28]. Issues related to semantic validation and understanding identified in the initial Chinese version were addressed after receiving feedback from the expert review and cognitive interviewing, leading to improvements such as clarifying complex terms and vague definitions. Item analysis revealed good differentiation and high correlations between the items in this study. Furthermore, this instrument underwent rigorous testing for internal consistency, test-retest reliability, and construct validity, exhibiting excellent internal consistency, time stability, and construct validity, resonating with the original study [19]. The types of reliability and validity assessed in this study represent essential psychometric properties for a measurement instrument.

Discrepancies between existing instruments largely stem from differing conceptual frameworks. The DHLI measures 7 individual skills: operation, navigation, information searching, evaluating reliability, determining relevance, adding self-generated content, and protecting privacy [16]. The eHLQ comprises 7 dimensions, addressing users’ attributes, their interaction with technologies, and their experience with systems [17]. Our CFA findings confirm a 5-factor structure, consistent with the exploratory factor analysis findings of the initial study [19]. Beyond competence in using and evaluating digital solutions and ICTs, the DigiHealthCom instrument addresses additional essential domains that were previously unaddressed, that is acceptance of digital solutions, human-centered remote counseling, digital interaction skills with patients and interprofessional teams, and ethical competence related to digital solutions [19]. Notably, acceptance of digital solutions significantly influences health care professionals’ adoption of digital solutions [13,14]. Competence in person-centered remote consultations is crucial for fostering patient engagement and improving accessibility to equitable digital health services [8]. Furthermore, proficient digital interaction skills facilitate effective coordination in digital settings, thereby supporting decision-making and optimizing treatment plans [8]. As digital solutions become more prevalent, attention to data privacy and information security has intensified [29,30]. Ethical competence related to digital solutions ensures appropriate management of patient information, fostering trust in digital health technologies and enhancing the safety of digital health services [20,31]. These domains provide a unified theoretical framework for comprehensively measuring digital health competencies among various professionals [8,9,19], which are essential for delivering high-quality digital health services to meet future demands.

The Finnish health care system is renowned for its universality, robust public funding, and centralized digital infrastructure [32]. Regional variations exist in Finland; for example, northern districts have fewer professionals specializing in remote counseling and the integration of digital solutions compared with southern areas [20,33]. Strategies have been implemented to enhance health care professionals’ digital health competence and improve education related to health care digitalization, including technology to support client engagement, digital services integrated into nursing work, and considerations of safety and ethics in the digital environment [20,34]. In contrast, China experiences regional disparities in digital health infrastructure and access to digital health services, especially in rural areas, despite the rapid growth of digital health services [35]. In addition, system compatibility across hospitals remains an unsolved issue [35]. These factors may influence the development of digital health competence among health care professionals. A cross-sectional study conducted in a central Chinese province reported that 49.9% (1690/3386) of clinical nurses demonstrate low telehealth readiness [36]. Consequently, evaluating, addressing, and reducing regional disparities in health care professionals’ digital health competence is vital for promoting equity in health care service provision. Validating a comprehensive digital health competence measurement instrument for health care professionals is imperative to address challenges posed by diverse health care environments, such as China’s. The Chinese version of DigiHealthCom is now ready for application in a wide range of settings and various health care professionals. Researchers, educators, health care providers, and policy makers can use it to evaluate digital health competence among diverse health care professionals, and develop digital health training curricula and policies based on the assessment.

### Limitations

This study acknowledges several limitations. First, the primary participants were nurses. Future research should involve a broader spectrum of health care professionals. Second, although recruited from diverse health care institutions, the participants mainly came from southern China, which may introduce selection bias due to regional disparities in digital health infrastructure. To address this, participants were divided into 2 groups (Southern China versus Northern and Western China) for sensitivity analysis, and their DigiHealthCom scores were compared. We did not find significant differences in digital health competence between the 2 groups, indicating that selection bias is unlikely in the study sample. Future studies could benefit from employing a stratified sampling method. Third, the absence of a standardized method for assessing digital health competence among health care professionals hindered the evaluation of criterion validity. Assessments of digital health competence predominantly rely on subjective self-reports, which might be susceptible to response bias. However, relying exclusively on performance-based assessments or other objective measures to evaluate competence also presents challenges. Therefore, using a more holistic combination of methods could offer a viable solution. Future research should consider larger sample sizes and multicenter external evaluations to address these limitations.

### Conclusions

In conclusion, we have successfully translated, culturally adapted, and validated DigiHealthCom for Chinese health care professionals. Our findings demonstrate that the Chinese version of DigiHealthCom is a reliable and valid instrument for assessing digital health competence among these health care professionals.

## Supplementary material

10.2196/65373 Multimedia Appendix 1 Modified items of the Chinese version of the DigiHealthCom (Digital Health Competence) instrument after cultural adaption.

10.2196/65373 Multimedia Appendix 2 Comparison of DigiHealthCom (Digital Health Competence) scores between health care professionals in the Southern China group and in the Northern and Western China group.

10.2196/65373 Checklist 1 Checklist for Reporting Results of Internet E-Surveys (CHERRIES).

## Abbreviations

AVE: average variance extracted
CFA: Confirmatory Factor Analysis
CFI: Comparative Fit Index
CHERRIES: Checklist for Reporting Results of Internet E-Surveys
CR: composite reliability
DHLI: Digital Health Literacy Instrument
DigiHealthCom: Digital Health Competence
eHLQ: eHealth literacy questionnaire
HTMT: heterotrait-monotrait ratio
I-CVI: item content validity index
ICC: Intraclass correlation coefficient
ICT: information and communication technology
IFI: Incremental Fit Index
NDASS: Nursing Digital Application Skill Scale
RSMEA: root-mean-square error of approximation
S-CVI/Ave: Scale CVI/Average
SRMR: standardized root-mean-square residual
TLI: Tucker-Lewis Index
WHO: World Health Organization

## References

[1] World Health Organization (2021). Global strategy on digital health 2020-2025. https://www.who.int/publications/i/item/9789240020924.

[2] World Health Organization (2019). WHO guideline recommendations on digital interventions for health system strengthening. https://iris.who.int/handle/10665/311980.

[3] Qin S, Zhang J, Sun X (2024). A scale for measuring nursing digital application skills: a development and psychometric testing study. BMC Nurs.

[4] Grand View Research Digital health market size, share & trends analysis report by technology (healthcare analytics, mhealth), by component (hardware, software, services), by application, by end-use, by region, and segment forecasts, 2024 - 2030. https://www.grandviewresearch.com/industry-analysis/digital-health-market.

[5] Nazeha N, Pavagadhi D, Kyaw BM, Car J, Jimenez G, Tudor Car L (2020). A digitally competent health workforce: scoping review of educational frameworks. J Med Internet Res.

[6] Odendaal WA, Anstey Watkins J, Leon N (2020). Health workers’ perceptions and experiences of using mHealth technologies to deliver primary healthcare services: a qualitative evidence synthesis. Cochrane Database Syst Rev.

[7] Bichel-Findlay J, Koch S, Mantas J (2023). Recommendations of the International Medical Informatics Association (IMIA) on Education in Biomedical and Health Informatics: Second Revision. Int J Med Inform.

[8] Jarva E, Oikarinen A, Andersson J (2022). Healthcare professionals’ perceptions of digital health competence: a qualitative descriptive study. Nurs Open.

[9] Konttila J, Siira H, Kyngäs H (2019). Healthcare professionals’ competence in digitalisation: a systematic review. J Clin Nurs.

[10] Brown J, Pope N, Bosco AM, Mason J, Morgan A (2020). Issues affecting nurses’ capability to use digital technology at work: an integrative review. J Clin Nurs.

[11] Kruk ME, Gage AD, Arsenault C (2018). High-quality health systems in the Sustainable Development Goals era: time for a revolution. Lancet Glob Health.

[12] Salahuddin L, Ismail Z (2015). Classification of antecedents towards safety use of health information technology: a systematic review. Int J Med Inform.

[13] Wernhart A, Gahbauer S, Haluza D (2019). eHealth and telemedicine: Practices and beliefs among healthcare professionals and medical students at a medical university. PLoS ONE.

[14] Alam K, Mahumud RA, Alam F, Keramat SA, Erdiaw-Kwasie MO, Sarker AR (2019). Determinants of access to eHealth services in regional Australia. Int J Med Inform.

[15] Longhini J, Rossettini G, Palese A (2022). Digital health competencies among health care professionals: systematic review. J Med Internet Res.

[16] van der Vaart R, Drossaert C (2017). Development of the Digital Health Literacy Instrument: Measuring a Broad Spectrum of Health 1.0 and Health 2.0 Skills. J Med Internet Res.

[17] Kayser L, Karnoe A, Furstrand D (2018). A multidimensional tool based on the eHealth Literacy Framework: development and initial validity testing of the eHealth Literacy Questionnaire (eHLQ). J Med Internet Res.

[18] Cowan DT, Norman I, Coopamah VP (2005). Competence in nursing practice: a controversial concept--a focused review of literature. Nurse Educ Today.

[19] Jarva E, Oikarinen A, Andersson J, Tomietto M, Kääriäinen M, Mikkonen K (2023). Healthcare professionals’ digital health competence and its core factors; development and psychometric testing of two instruments. Int J Med Inform.

[20] Jarva E, Oikarinen A, Andersson J, Pramila-Savukoski S, Hammarén M, Mikkonen K (2024). Healthcare professionals’ digital health competence profiles and associated factors: a cross-sectional study. J Adv Nurs.

[21] Cruchinho P, López-Franco MD, Capelas ML (2024). Translation, cross-cultural adaptation, and validation of measurement instruments: a practical guideline for novice researchers. J Multidiscip Healthc.

[22] Wenjuanxing. wjx. https://www.wjx.cn.

[23] Zhang C, Yang Z, Zhang H (2022). Psychometric evaluation of the Chinese version of occupational lowback pain prevention behaviors questionnaire among clinical nurses: a validation study. Front Public Health.

[24] Polit DF, Beck CT (2006). The content validity index: are you sure you know what’s being reported? Critique and recommendations. Res Nurs Health.

[25] Jalali A, Naghibzadeh A, Mohammadi MM (2024). Translation and validation of the Persian version of the Nursing Practice Readiness Scale (NPRS) for new graduate nurses. BMC Nurs.

[26] El-Ammari A, El Malki H, Moutawakkil SG (2023). Validation of the Center for Epidemiologic Studies Depression Scale (CES-D) in a Moroccan sample with substance use disorder. BMC Psychiatry.

[27] Eysenbach G (2004). Improving the quality of Web surveys: the Checklist for Reporting Results of Internet E-Surveys (CHERRIES). J Med Internet Res.

[28] Alelayan H, Liang L, Ye R, Aldosari N, Liao X (2022). Translation and linguistic validation of the DISABKIDS chronic generic module into simplified Chinese (DCGM-37) for use among children with cancer. J Spec Pediatr Nurs.

[29] Kaihlaniemi J, Liljamo P, Rajala M, Kaakinen P, Oikarinen A (2023). Health care professionals’ experiences of counselling competence in digital care pathways - a descriptive qualitative study. Nurs Open.

[30] Nittari G, Khuman R, Baldoni S (2020). Telemedicine practice: review of the current ethical and legal challenges. Telemed J E Health.

[31] Kaihlanen AM, Elovainio M, Virtanen L (2023). Nursing informatics competence profiles and perceptions of health information system usefulness among registered nurses: A latent profile analysis. J Adv Nurs.

[32] Kaihlanen AM, Virtanen L, Buchert U (2022). Towards digital health equity - a qualitative study of the challenges experienced by vulnerable groups in using digital health services in the COVID-19 era. BMC Health Serv Res.

[33] Ahonen O, Kouri P, Salanterä S, Liljamo P, Kinnunen UM, Saranto K Finnish nurses association’s digital social and health services strategy. Finnish Nurses Association.

[34] Tynkkynen LK, Karanikolos M, Litvinova Y (2023). Finland: Health system summary.

[35] Wu G, Gong M, Wu Y, Liu L, Shi B, Zeng Z (2024). Advancing digital health in China: aligning challenges, opportunities, and solutions with the Global Initiative on Digital Health (GIDH). Health Care Sci.

[36] Yu-Tong T, Yan Z, Zhen L, Bing X, Qing-Yun C (2022). Telehealth readiness and its influencing factors among Chinese clinical nurses: a cross-sectional study. Nurse Educ Pract.

