# Supplementary material for: The Chinese Version of the DigiHealthCom (Digital Health Competence) Instrument for Assessing Digital Health Competence of Health Care Professionals: Translation, Adaptation, and Validation Study
Source: JMIR Hum Factors. 2025 Mar 21;12:e65373. doi: 10.2196/65373 (PMC11951814; doi:10.2196/65373)
Supplement: Multimedia Appendix 1 [file humanfactors-v12-e65373-s001.pdf]

Table S1. Modified items of the Chinese version of the DigiHealthCom instrument after cultural adaption.

Item	Initial version	Final version	Reasons for revision
[a]RC1: I can act in reciprocal (aiming towards respect and equality) interaction with the customer in remote counselling	我能在远程咨询中与客户/患者互惠（旨在尊重和平等）互动。	我能在远程咨询中与客户/患者相互尊重和平等互动	One nurse indicated that “reciprocal interaction” was difficult to understand. Therefore, we used "mutual respect and equality interaction" to express “reciprocal interaction”.
RC8: I can guide the customer verbally in remote counselling (e. g. on the phone without video)	我能在远程咨询中口头指导客户/患者（例如：使用不带视频的电话）	我能在远程咨询中通过语言指导客户/患者（例如：在没有视频的情况下进行电话咨询）	One expert recommended using a more specific example, such as “conducting telephone consultations without video.”
RC16: I can guide the customer to find reliable information (e. g. from the Finnish Institute for Health and Welfare, Social Insurance Institution of Finland, Health Village, Health Library, Nursing Research Foundation)	我能指导客户找到可靠的信息（例如：从健康教育网、国家医保服务平台等）	我能指导客户/患者找到可靠的信息（例如：从具有公信力的健康教育网站）	One expert indicated that the examples in the item were not common in China. It was recommended to modify to read “from reputable health education websites.”
[b]DS7: Digital services are a good way to deliver social and health services (e. g. customer work, care, rehabilitation)	数字化服务是一种提供社会和健康服务（例如：客户工作、照护、康复）的好方式	数字化服务是一种提供社会和卫生健康服务的好方式（例如：客户服务、医疗、康复）	One expert indicated that the example “customer work” in the item was not common in China and should be replaced semantically with “customer service”.
[c]UE3: I can recognise factors (e. g. resources, motivation) that influence the utilisation of digital solutions	我能识别影响数字化解决方案使用的因素（例如：资源、动机）	我能识别应用数字化解决方案的影响因素 （例如：数字资源、使用动机）	One expert indicated that the examples in the item might be difficult to understand. It was recommended to modify to read “digital resources, use motivation.”

[a]RC: human-centered remote counseling competence.

[b]DS: digital solutions as part of work.

[c]UE: competence in utilizing and evaluating digital solutions.
