# Supplementary material for: The Chinese Version of the DigiHealthCom (Digital Health Competence) Instrument for Assessing Digital Health Competence of Health Care Professionals: Translation, Adaptation, and Validation Study
Source: JMIR Hum Factors. 2025 Mar 21;12:e65373. doi: 10.2196/65373 (PMC11951814; doi:10.2196/65373)
Supplement: Multimedia Appendix 2 [file humanfactors-v12-e65373-s002.docx]

**Table S3.** Comparison of DigiHealthCom scores between health care professionals in the Southern China group and in the Northern and Western China group.

| Dimensions | Southern China  (N=351) | Northern and Western China  (N=47) | *t* value | *Ρ* |
| --- | --- | --- | --- | --- |
| RC^a^ | 3.26 (0.56) | 3.20 (0.52) | 0.67 | .506 |
| DS^b^ | 3.41 (0.58) | 3.49 (0.53) | -0.94 | .346 |
| ICT^c^ | 3.46 (0.61) | 3.49 (0.58) | -0.37 | .710 |
| UE^d^ | 3.23 (0.61) | 3.24 (0.61) | -0.06 | .950 |
| EC^e^ | 3.31 (0.62) | 3.27 (0.66) | 0.36 | .720 |
| Total | 3.31 (0.53) | 3.31 (0.48) | 0.02 | .981 |
| ^a^RC: human-centered remote counseling competence.  ^b^DS: digital solutions as part of work.  ^c^ICT: information and communication technology competence.  ^d^UE: competence in utilizing and evaluating digital solutions.  ^e^EC: ethical competence related to digital solutions. | | | | |
